# Lunatic Asylums and Insanity in America

**Published:** 1849-07-01

**Authors:** 


ART. IV.—*The American Journal of Insanity.* Edited by the Officers of the New York State Lunatic Asylum, Utica. Vols. I. to V. Printed at the Asylum. 1849.

THE Americans are an energetic, active people. The rapidity of their progression in all the arts connected with civilization is one of the marvels of modern history. The metropolis of the New World vies in wealth, luxury, and magnificence with the metropolis of the mother country. In science and literature the same enterprising spirit is abroad; and philosophical inquiries are conducted with an earnestness and zeal, and with an independence of principle, in the highest degree favourable to the revelation of truth. Here the field of speculative philosophy is not encumbered with scholastic dogmas thrown like brambles across our path. No ancient prejudices fetter our inquiries. Men of science associate together to promote the progress of knowledge, and rejoice in breathing an air of intellectual freedom. The American mind is essentially independent. There are no university traditions which cloud its vision; and as nature is the same now as it was the first morning of creation, the absence of ancient authorities prescribing empirical forms of belief, is rather favourable than otherwise to the progress of mental philosophy. The zeal with which speculative science is being pursued in America is highly characteristic of this state of progression—nay, in psychology and the department of medical science to which this journal is specially devoted, there is an activity displayed which has not yet appeared on this side of the Atlantic. Already an association analogous to the Psychological Society of Paris has been formed in America. The medical superintendents of the different institutions for the insane have united and formed an association, which holds meetings at stated periods, when papers are read and reports made on all subjects connected with insanity.

On the 8th of May, 1848, the third meeting of this association took place at Astor House, in the city of New York, when, we are informed, written reports were made on the following subjects, and after full discussion, accepted and laid upon the table, subject to future disposition by the association—viz.,

"On the comparative value of the different kinds of Labour for Patients, and the best means of Employment in Winter, by Dr. Rockwell."

"On the advantages and disadvantages of Cottages for wealthy Patients adjacent to Hospitals for the Insane, by Dr. Kirkbride."

"On the relative value of the different kinds of Fuel for heating hospitals, by Dr. Bates."

"On the most economical mode of treating the Insane of the Poorer Classes, by Dr. McFarlane."

"On Reading, Recreations, and Amusements for the Insane, by Dr. Galt."

"On the comparative value of Treatment in Public Institutions and Private Practice, by Dr. White."

"On the use and effects of Tobacco on the Insane, by Dr. Cutter."

It is also stated, that in conformity with a resolution adopted at the last meeting of the association, Drs. Brigham and Macdonald made written, and Drs. Earle, Rockwell, Bates, Butler, Allen, and Kirkbride, verbal reports on the subjects of post-mortem examinations and the pathology of insanity, which were referred to the standing committee on these subjects. On the 21st of May, 1849, another meeting of this association was held, the transactions of which have not yet reached us, but we refer to the fact of such a society existing in America as an evidence of the activity and energy with which our Transatlantic brethren are pursuing these inquiries.

The American Journal of Insanity, now upon our table, supplies a further testimony of the zeal evinced in this department of medical science. It is under the editorship of Dr. Brigham, assisted by the principal officers of this association, and is published quarterly. It contains original articles on psychology and mental pathology, selections carefully and judiciously made from contemporary journals, and reports on the state of lunacy generally, in America and other countries. The number of institutions open for the reception of the insane in the United States was, in July last, thirty. Fifteen of these are state institutions, governed by a board of managers, or a committee appointed by the state. Five are corporate institutions in connexion with general hospitals, constituting the insane department of such establishments, and governed by the same authorities. Five are institutions that have been established by the liberal benefactions of individuals, and are governed by a board of directors appointed by the donors and their successors. There are also three private asylums, and some of the states are about to open similar institutions. The returns, however, appear at present to be limited to thirty asylums; but insane patients are received in several private families, and many are kept in town or country alms-houses in all the states of the Union. The number of the insane in the United States does not appear to be satisfactorily ascertained. In 1840, the insane and idiotic in the state of New York were 2340, and five years later, according to the census of 1845, there were 3752, or 2142 lunatics and 1610 idiots.

There are, it is presumed, at the present period at least 18,000 insane persons in the United States, not including idiots, which are supposed to be 6000, so that we have from the statistics before us an aggregate of 24,000 lunatics and idiots, and yet the various institutions in the United States will not accommodate more than 4711 patients.

On this side of the Atlantic, the Commissioners in Lunacy in their last report estimated the aggregate number of the insane and imbecile, in England and Wales, together with their various committees, visitors, medical officers, attendants and servants, at not less than 30,000, and the number of establishments, public and private, at 614, independent of 596 parish workhouses, in which many of the insane and imbecile poor are located. The present inadequacy of the institutions for the insane in the United States has called forth the strongest and most energetic appeals and remonstrances from the association of medical officers, who are so creditably and honourably engaged in this department of the profession. The system of domiciling lunatics and idiots in workhouses is universally condemned; and we believe both here and in America it has been had recourse to only in cases of absolute necessity. We would, however, deprecate in a still stronger manner the admission into any institution for the insane of patients affected with other diseases. From the reports of the Mount Hope Institution, we find cases admitted into that establishment of delirium tremens, erysipelas, syphilis, cancer, typhus fever, &c., and as there is but one building, the persons afflicted with these loathsome diseases are in the same rooms with the quiet and convalescent insane. Furthermore, this institution is not, we are informed, subject to legal visitation or regular inspection by the authorities of the state. The proprietors manage it as they choose, without being called upon to give any account of their proceedings. We cordially agree with the editor of this journal, that such an institution is an anomaly, and ought not to be permitted. We also fully concur with him as to the necessity of there being a resident medical officer in every institution for the cure of the insane. "At a time when it was supposed that all that could be done for the welfare of the insane was to keep them closely confined, so that they could not injure themselves or others, it was not deemed necessary to have a physician or resident officer on the building appropriated to them; but of late years, since insanity has been deemed a curable disease, the opinion is entirely the reverse. Hence all modern, well-conducted institutions for the insane have one or more medical officers. This is so obviously proper, so necessary to the safety of patients, in case of accidents or sudden change in their condition, so requisite for observing their habits and varying states of mind and body, in order to treat them properly, that we presume no one will venture to dispute it." While it may be the more pleasing task of the editor of such a journal as the one before us to record the progress of this branch of medical science, it is clearly a duty he owes to society to call the attention of the legislature to these and all other abuses in such institutions, with a view to their immediate correction. It should be remembered, that the victim of insanity has the strongest claims upon the sympathies of humanity; he is often incapable of describing his misery, or even complaining of the ill-treatment to which he may be subjected; and hence in every civilized country, it is the duty of the legislature to throw a protection round these dependent and helpless beings.

It is true the unhappy lunatic is no longer left, as in the time of the Jews, to wander and dwell amidst the tombs, or lie down as the "impotent lay at the pool of Siloam." Institutions, founded on the most philanthropic principles, have been established in every Christian land—

hospitals for the sick—asylums for the insane—houses of refuge for the destitute. The stream of Christian charity flows silently, but deeply fertilizing and blessing the soil through which it extends. In no instance has this principle of philanthropy been more beautifully exemplified than in the success which has crowned the attempts which have been made in France, Prussia, Switzerland, and America, to ameliorate the mental condition of the demented, the imbecile, and the idiotic. In 1846, commissioners were appointed by the Legislature of Massachusetts, "to inquire into the condition of the idiots of the commonwealth, to ascertain their number, and whether anything could be done for their relief."

In 1847, Dr. S. G. Howe reported:—

"We have obtained pretty satisfactory information from 171 towns, containing an aggregate population of 345,285 inhabitants. From these towns we have reports containing the names, age, sex, condition, &c., of 543 idiots, 204 of whom are males, and 339 are females. Of these, 169 are less than 25 years of age, and of course are proper subjects for instruction. Of the whole number, 106 are supported entirely at public charge. If the other towns should present the same number, it would show an aggregate of over 1000 idiots in this commonwealth, of whom 300 are of proper age for instruction. We have also obtained information, by personal inspection of the idiots, in about 30 towns, in various parts of the state, which shows that the condition of these unfortunate persons is very materially influenced by the character of those who have the charge of them. In some towns, we found the idiots, who were under the charge of kind-hearted, but ignorant persons, to be entirely idle, given over to disgusting and degrading habits, and presenting the sad and demoralizing spectacle of men, made in God's image, whom neither their own reason, nor the reason of others, lifted up above the level of the brutes. In other towns, idiots, who to all appearance had no more capacity than those just mentioned, were under the charge of more intelligent persons, and they presented a different spectacle—they were healthy, cleanly, and industrious. We found some, of a very low grade of intellect, at work in the fields, under the direction of attendants; and they seemed not only to be free from depraving habits, but to be happy and useful. The inference to be drawn from this is very important. If persons having only common sense and common humanity, but without the advantage of experience or study, can so improve the condition of idiots, how much could be done by those who should bring the light of science, and the experience of wise and good men in other countries, and the facilities of an institution adapted to the training of idiots,—how much, we say, could be done by such persons, towards redeeming the minds of this unfortunate class from the waste and desolation in which they now lie!"

To this report, Dr. Howe has appended the following interesting remarks, from a letter addressed to him by Mr. George Sumner from Paris:—

"During the past six months, I have watched, with eager interest, the progress which many young idiots have made, in Paris, under the direction of Mr. Seguin, and at Bicêtre, under that of Messrs. Voisin and Vallee, and have seen, with no less gratification than astonishment, nearly one hundred fellow-beings, who, but a short time since, were shut out from all communion with mankind,—who were objects of loathing and disgust,—many of whom rejected every article of clothing,—others of whom, unable to stand erect, crouched themselves in corners and gave signs of life only by piteous howls,—others, in whom the faculty of speech had never been developed,—and many, whose voracious and indiscriminate gluttony satisfied itself with whatever they could lay hands upon—with the garbage thrown to swine, or with their own excrements; these unfortunate beings—the rejected of humanity—I have seen properly clad, standing erect, walking, speaking, eating in an orderly manner at a common table, working quietly as carpenters and farmers; gaining by their own labour, the means of existence; storing their awakened intelligence by reading one to another; exercising, towards their teachers and among themselves, the generous feelings of man's nature, and singing, in unison, songs of thanksgiving!"—*Journal of Insanity*, vol. iv. p. 79.

From the period when this commission was appointed by the Legislature, April 11th, 1846, this benevolent object has been proceeded with; and more suitable accommodation has been provided for the insane and idiotic at Massachusetts, than in any other state in the Union. In the last number of the Journal before us, (April, 1849,) we find the following very interesting and satisfactory report on the condition and capacities of the idiots in Massachusetts:—

"'Confining our attention to the cases of real idiots, above mentioned—viz., 420 out of 574—it is found that 188 are under 25 years of age. Of these, 172 seem capable of improvement; they present proper cases for attempts at instruction, and the formation of regular, industrious, and cleanly habits. Only 16 seem incapable of improvement. Of those over twenty-five years of age, there are 73 who seem capable of little or no improvement in mental condition. Of the 420 idiots proper, 19 can now earn their board and clothing, under the management of discreet persons; 141 do earn their board, when properly managed; 110 can do trifling work, if carefully watched and directed; 73 are as helpless as children of seven years old; 43 are as helpless as children of two years old; and 34 are as utterly helpless as infants.

"'With regard to pecuniary circumstances, 20 have property of their own, held by guardians; 26 belong to wealthy families; 196 belong to indigent families, but are not public paupers; 148 are town or state paupers; the rest are sometimes aided by the public, sometimes not. Of the whole number—viz., 574—there are 220 at town or state charge.

"'Of the 420 idiots proper, 218 are insatiable gluttons; and 102 are known to be given to self-abuse in a frightful degree.'

"After describing their wretched condition, and also the successful efforts made in Europe for the physical improvement and education of this class of persons, the commissioners strongly urge that '*measures be at once taken to rescue this most unfortunate class from the dreadful degradation in which they now grovel;*' and add, 'Massachusetts admits the right of all her citizens to a share in the blessings of education, and she provides it liberally for all her more favoured children. If some be blind or deaf, she still continues to furnish them with special instruction at great cost; and will she longer neglect the poor idiot—the most wretched of all who are born to her—those who are usually abandoned by their fellows—who can never, of themselves, step upon the platform of humanity—will she leave them to their dreadful fate, to a life of brutishness, without an effort in their behalf?

"'It is true, that the plea of ignorance can be made in excuse for the neglect and ill-treatment which they have hitherto received; but this plea can avail us no longer. Other countries have shown us that idiots may be trained to habits of industry, cleanliness, and self-respect; that the highest of them may be measurably restored to self-control, and that the very lowest of them may be raised up from the slough of animal pollution in which they wallow; and can the men of other countries do more than we? Shall we, who can transmute granite and ice into gold and silver, and think it pleasant work—shall we shrink from the higher task of transforming brutish men back into human shape? Other countries are beginning to rescue their idiots from further deterioration, and even to elevate them; and shall our commonwealth continue to bury the humble talent of lowly children committed to her motherly care, and let it rot in the earth, or shall she do all that can be done to render it back with usury to Him who lent it? There should be no doubt about the answer to these questions. The humanity and justice of our rulers will prompt them to take immediate measures for the formation of a school or schools for the instruction and training of idiots.'

"Subsequently, during the latter part of the session of 1848, 'the Legislature of Massachusetts made an appropriation of $2500 per annum, for three years, to be devoted to the experiment of teaching and training ten idiots.

"A school has been established at South Boston, under the direction of Dr. Howe; and several idiots are already under instruction."—*Journal of Insanity*, vol. v. pp. 374, 375.

In the fourth volume of the journal, Dr. Earle, physician to the Bloomingdale Asylum, contributes a valuable article on the causes of insanity. Upon the obscure subject of hereditary transmission, direct and indirect, he observes—

"During the first few years of the existence of the asylum, there appears to have been but little attention paid to this particular subject, and hence the records thereupon are imperfect. There are other important obstacles in the way, to a correct knowledge of the full extent of which the hereditary predisposition prevails among the patients admitted into a public institution. These obstacles may, by perseverance, be measurably overcome.

"Insanity being a disordered manifestation of the mind, dependent upon some disease of the body, either functional or organic, is subject to the same laws as many or most other maladies to which the human race is subject. Like consumption, gout, diseases of the liver and of the heart, it may attack any person whatever, but is certainly somewhat more likely to prevail among those whose ancestors have suffered from it.

"Of the men included in the foregoing table, 118 inherited the predisposition from direct ancestors, and 33 of these had other relatives insane. The remaining 58 had collateral relatives insane, but no direct ancestors. Of the 52 who had insane parents, it was the father in 27 cases, and the mother in 25. In one of these, both father and mother had been deranged. It is also stated, that two of those included under the term hereditary had ancestors, both paternal and maternal, who were subject to the malady, and one who had a daughter insane.

"Of the women, the predisposition was transmitted from direct ancestors in 89; of whom 67 also had other relatives insane. In the remaining 42, the disease is stated to have appeared only in persons collaterally connected, and in five cases in their children alone. There are 18 cases in which it is mentioned that the father was insane. In one case, the father and mother were both deranged. In the case where it is mentioned that the whole family were insane, it is said that all her father's family, which consisted of 12 children, have been insane, and that their insanity did not, in a single instance, make its appearance before the age of 21 years. Two of her brothers, while insane, committed suicide. None of the third generation have yet been attacked with insanity, although several of them have passed the age at which it made its appearance in the second."

Dr. Earle next enters upon the physical and moral causes of insanity, and out of 1186 cases, 664 are ascribed to physical, and 522 to moral causes. The older authors entertained the opinion that mental causes were more prolific of insanity than physical causes. "Within a few years, however, the opposite opinion has been gaining ground—an opinion" says Dr. Earle, "which is sustained by these statistics." But we may here observe that statistical inductions, unless founded upon sufficient data, must always be unsatisfactory. There is no branch of science in which we are so liable to be deceived as statistics; for unless the aggregate number of cases included in the induction are sufficient to represent the universality of nature in precisely analogous conditions, we are only misled by calculating them. Among the physical causes of insanity, intemperance, according to Dr. Earle, ranges highest; and yet may not the intemperance itself be considered more properly as an effect or result of moral insanity? Among the moral causes, pecuniary difficulties range highest; yet may not this be mixed up with such other moral causes as mental excitement, domestic trouble, anxiety, mortified pride, disappointment, each of which has a different and separate position in Dr. Earle's scale of causation? There is an old aphorism of Hippocrates, that no disease is produced by a single cause; and upon this point we find our nosologists generally at fault. Among the physical causes of insanity, thirteen cases are supposed to have resulted from the excessive use of opium; and Dr. Earle appears to think that tobacco, when used by smoking, may tend to disturb the functions of the liver, and by disordering the action of this organ, become a not unfrequent cause of mental disease. To this opinion we can hardly subscribe; at all events, the Turks, who smoke opium, and the Germans, who are not only inveterate smokers of tobacco, but of *bad* tobacco, are not peculiarly liable to insanity. In many instances that have come under our observation, smoking tobacco has had a comforting and soothing effect, as Dr. Earle admits in the following remarks:—

"How little or how much soever tobacco may act, either immediately or remotely, as a generative cause of insanity, it is a fact well known to all connected with public institutions of this kind, that there is no stimulus or narcotic substance in which the insane are more prone to indulge. If within their reach, those who, previously to becoming insane, have been accustomed to it, will use it to excess, and many or most of those who have not before been addicted to the habit, soon become accustomed to it. One man, included among the patients remaining in the institution at the time these statistics close, kept constantly in his mouth, both day and night, excepting when at meals, a quid of tobacco, frequently as large as an ordinary hen's egg. Whatever saliva it might have produced, it was rarely, if ever, ejected from the mouth, but usually swallowed. He had been in the institution during the whole period of its existence, being one of those who were brought from the old asylum. He had been accustomed to the habit for many years; and it might also be said of him that—

"Like to the Pontic monarch of old days,
He fed on poison, and it had no power,
But was a kind of nutriment."

"Although as completely insane and incoherent as it is possible for a human being to be, he worked regularly, doing about as much as any ordinary labourer. The tobacco appeared to have a soothing and controlling effect upon him, enabling him to concentrate his powers upon the labour in which he was employed. If deprived of it for a few hours, he became restless, agitated, excited, talkative, and unable to apply himself to his occupation. In this respect, the narcotic had an opposite effect upon him to that which it produces upon many of the insane. It frequently increases their excitement, and, in some instances, to a remarkable degree. Its action, upon the whole, is considered so deleterious, that in most of the well-conducted establishments for the insane in this country, its use among the patients is prohibited. At this institution, it is not permitted, excepting in a few cases, in small quantities, by patients who have resided here many years."

There is one cause of insanity, mentioned by Dr. Earle, which we do not before remember to have met with — viz., mesmerism. Deleuze, Dupotet, Elliotson, Colquhoun, and other champions of this subtle art, insist that the practice of mesmerism is never followed by any permanently unhappy or fatal result. The subjoined case, however, would seem to establish that insanity may be produced by this cause:—

"The patient was a young man, about twenty years of age, of a highly nervous temperament, with a brain remarkably developed, and corresponding intellectual powers. For several years he had suffered from occasional epileptic fits, which, as yet, had left his mind but little if at all impaired. The skill of many physicians, and the virtues of every medical resource, believed to be applicable to such cases, had been exhausted upon him without benefit. As a dernier resort, and at a period when he was in a state of comparative stupor, such as frequently follows a succession of epileptic fits, he was placed under the care of a person professedly practising 'mesmerism' for the cure of disease. To use the expression of this person, 'The patient was magnetized daily, for nearly a month,' without effect, he remaining in the torpid condition already mentioned. At length, he was suddenly roused, appeared rational for a few hours, and then passed into a state of high excitement and absolute mania. A day or two afterwards, he was brought to the asylum, with his arms and legs strongly bound. When admitted, he talked but little, and that little was perfectly devoid of meaning. He was highly excited, his face flushed, and the veins of his head swollen; the circulation rapid, the pulse being from one hundred and twenty to one hundred and forty per minute, the tongue furred, and the bowels very much constipated. After free catharsis, an inordinate quantity of medicine being required to operate upon his bowels, he was placed upon the use of sedatives. Under this treatment, and after the lapse of two days, he began to improve, and in eight days he left the asylum, restored to his ordinary condition, and without so much of the torpor as existed previously to his excitement."

In the same volume, Dr. Ray, the superintendent of the Butler

Hospital for the insane, contributes a very interesting article on the legislation for the insane in the Maine. The legislature of the Maine passed, it appears, during the last session, an act for regulating the government and management of the insane, which is highly creditable to the humanity and judgment of that body. The mode of determining the admission of the insane into asylums is very simple, and similar to the plan prescribed by the code in France.

"The act provides, that on the application of any relative of an insane person, or any justice of the peace, the mayor and aldermen of cities, and the select men of towns, shall examine into the case of such insane person, and if satisfied that the person is insane, and that 'his comfort and safety, or those of others interested, would be promoted by a residence in the insane hospital,' shall send him forthwith to the hospital, where he must stay if the superintendent see fit to keep him, at least six months. If their decision is not satisfactory to any of the parties interested, an appeal is provided to certain justices of the peace, who institute a new and final trial of the case."

The Avocat does the duty in Paris which the justice of the peace, or mayor, is here called upon to perform; and the obvious advantage of the plan is, that the party who adjudicates upon the insanity of the person has no interest whatever in his detention. Another remarkable part of this act of the Maine is, that it provides a change in the ordinary methods of criminal procedure in cases where the accused is alleged to be insane.

"'When any person,' says the act, 'shall be charged with a criminal offence in this state, any judge of the court before which he or she is to be tried, on notice that a plea of insanity will be made, or when such plea is made in court, may, if he deem proper, order such person into the custody of the superintendent of the insane hospital, to be by him detained and observed, until the further order of the court, in order that the truth or falsehood of the plea may be ascertained.' This course, virtually, is pursued in France and most of the German states. It is unknown, however, to the forms of the English common law, and this, we suspect, is the first attempt to incorporate it with those forms. We apprehend no difficulty whatever in the practical working of this provision, and we anticipate as its certain result, that the ends of justice will be more effectually obtained, and the common prejudice against the plea of insanity in criminal cases be removed. We cannot better express our own views on this point than by quoting what we have already said in another place—

"'A very serious evil in the administration of the criminal law in cases where insanity is pleaded in defence, is the absence of any legal provision for satisfactorily establishing or disproving its existence. The matter is left entirely to the counsel, who use such means as they please and the law permits. They summon only such witnesses as suit their purposes; and medical men can generally be found—we regret to say it—ready to testify for or against the insanity of the accused, who have had but little practical knowledge of the disease, and have made but a superficial examination of the case in hand. Witnesses summoned in this manner will be liable, in spite of themselves, to testify under a bias, instead of expressing the results of a dispassionate examination of scientific facts. The intention of the prisoner's counsel to plead insanity may not be known to the government-counsel in season to meet the plea with appropriate evidence; and if the prisoner is acquitted, the impression is conveyed, that the ends of justice have been defeated. Indeed, with every disposition to arrive at the truth, it is generally impossible under the present arrangements. In gaols, where prisoners accused of crime are confined, proper opportunities are not afforded for investigating their mental condition. In the few formal interviews to which the observation of the prisoner is confined, it may often happen that the real condition of the mind will not be discovered. If really insane, he will be likely to control his movements, and to discourse and appear very differently from what he would when left to himself and unconscious of being observed. Many insane, as we have already shown, manifest their aberration only under certain circumstances, and on particular occasions, and appear quite correct at all other times. Many, too, whose insanity is recognised by everybody who knows them, never evince it in their discourse, but solely in their ways and habits. If, on the other hand, the prisoner is feigning insanity, he will summon all his powers to produce the requisite impression at these interviews, which being short and few, the difficulty of his task is much lessened. To ascertain satisfactorily the mental condition of a prisoner suspected of being insane, he should be placed where the expert may be able to see him often, and at times when he is not aware of being observed. His words, and acts, and movements, his manners and habits, should be systematically watched; and a single day of such observation would often throw more light on the case than many formal interviews. We see no difficulty in so changing our modes of criminal procedure, that when the court shall be satisfied that there are reasonable doubts of the prisoner's sanity, it may be authorized to postpone the trial, and place him, in the meantime, in the charge of an expert—for which our hospitals for the insane furnish a convenient and suitable opportunity—whose report shall be received in evidence at the trial. This is substantially the course adopted in France, and nothing short of its adoption with us will render the plea of insanity powerless for evil, and remove the suspicions of the community upon this point."—*Journal of Insanity*, vol. iv., pp. 215, 216.

Another important provision, which does infinite honour, says Dr. Ray, truly, to the humanity and intelligence of the legislature of the

Maine is, that "no insane person shall be committed to, or remain in, any gaol or house of correction;" and that, "when any inmate of the state prison becomes insane, a commissioner shall be appointed by the governor to examine his case, and if he be found insane, he shall be sent to the insane hospital. We hope this noble example will be speedily followed; and that, in New England at least, the confinement of the insane in gaols will be remembered as among the things that are passed."

There is no subject more difficult to speculate upon than the mortality of the insane; because patients who are admitted into lunatic asylums are often the subjects of other diseases which may prove fatal. Organic affections of the heart and lungs, stomach and liver, often co-exist with insanity, and are the ostensible cause of death. Upon this subject, we find in the volume before us the following pertinent observations:—

"The mortality of the insane, though an interesting subject, is one difficult to study with the accuracy requisite to satisfactory results. It depends on such varying and local circumstances, that nothing conclusive is learned by comparing the number of deaths at one institution with those of another.

"Some asylums are able to select their cases, and rarely receive any very bad ones; others do not receive the epileptic insane and those disposed to suicide, unless provision is made by their friends for special attendance and care; while some institutions are obliged to receive all that are sent to them. Again, in some asylums, many of the patients are from the immediate neighbourhood, and are supported by their friends, and when likely to die, are removed to their homes, while most of those in other establishments have no friends to take care of them, or are from a distance too remote to be sent home when feeble.

"Pinel, setting aside cases of senile dementia, estimates the mortality of the insane at one to twenty or twenty-three. Raymond found the mortality at Marseilles to be as one to fourteen. Tenon, at Paris, in 1786, fixed it at one to eleven. Esquirol thought it higher, even one to six or eight, and gives the following from his records:—

"Mortality in mania, one to twenty-five.
" " monomania, one to sixteen.
" " lypemania, or melancholy, one to twelve.
" " dementia, one to three.

"According to the records of the lunatic asylums in the Northern States of this country, for the last five years—viz., in Maine, New Hampshire, Vermont, Connecticut, the McLean, South Boston, and Worcester, Massachusetts; the Bloomingdale and Utica, New York; the Friends' Asylum, and the Pennsylvania Hospital for the insane, Pennsylvania, and the Ohio, the mortality in none is higher than

*one in eleven.* Eight thousand seven hundred and twenty-four patients have been treated at these institutions during the last five years, and the deaths have been 687, or about one in thirteen.

"We have not complete returns from the asylums in the Southern States, but judging from those we have, their mortality is greater; but we shall endeavour to procure more full statistics on this subject, and recur to it again."

An article on the paralysis peculiar to the insane (*paralysie generale*) in the volume before us, also merits attention; but we can only afford space for the following introductory observations:—

"This singular affection has been well described by Esquirol, and more fully by Bayle, Calmeil and Guislain, and more recently by several other writers. It should not be confounded with ordinary paralysis that arises from cerebral hæmorrhage or from ramollisement or tumours of the brain. The *paralysie generale* seems to have a different cause, and to arise from a kind of chronic inflammation of the membranes of the brain that cover the superior parts of the brain. This form of paralysis is more frequent among insane men than women. 'Eighteen years ago,' says Esquirol, when charged with the service of the division of the insane at the Bicêtre, during the absence of M. Pariset, who was sent to Cadiz to study the yellow fever which was prevailing there, 'I was struck in comparing the number of men, insane and paralytic, in the Bicêtre, and the number of paralytic women at the Salpêtriere. The same observation may be made in every establishment into which both sexes are admitted. It has not escaped the notice of Dr. Foville, physician-in-chief at St. Yon, Rouen. According to this physician, they amount to one-eleventh at the institution over which he presides. Among 334 insane persons who were examined by him, 31 were paralytic—to wit, 22 men and 9 women. At Charenton, the proportion of paralytics is still more considerable. They constitute one-sixth of the whole number of admissions. In truth, of 619 insane persons who were admitted during the three years—1826, 1827, 1828—109 were paralytics. But the proportion of men is enormous compared with that of women. Of 366 insane men admitted into the house, 95 were paralytics; while of 153 women, 14 only were affected with paralysis. This complication is most frequently observed among that class of insane persons who have yielded to venereal excesses, or have been addicted to the use of alcoholic drinks; among those, also, who have made an inordinate use of mercury, as well as those who, exercising the brain too vigorously in mental strife, have, at the same time, abandoned themselves to errors of regimen.'

"These circumstances explain why it is that there are more insane and paralytic men than women, and why this disease is more frequently seen in asylums for the insane that are in the vicinity of large cities and receive the wealthy and dissipated, than in those remote from cities, and that are filled mostly by the poor and industrious. We think, also, it explains, what we believe to be facts, that there is less of this disease in this country than in Europe, and that it is on the increase. A most striking peculiarity generally noticed in this complaint is, that those affected by it entertain the most extravagant notions of their wealth, grandeur, and power, and do not appear to suffer in body or mind, but continue cheerful and full of hope until they die.

"The first published notice of this disease in this country was given by Dr. Bell, of the McLean Asylum for the insane, in his Annual Report for 1843. He says:—'That terrible complication of insanity termed *paralysie generale* by the French, and of which general paralysis can scarce be deemed a synonyme, since the impairment of the nervo-muscular apparatus forms by no means a prominent symptom as in ordinary paralytic affections, and, indeed, for a period in the progress of the malady, scarcely an appreciable manifestation, is one which presents a large proportion of cases in the insane hospitals of Europe.' "

There are many articles in the volumes before us of a strictly practical nature; the following observations on the effects of the inhalation of sulphuric ether, in cases of insanity, will be read with interest:—

"We have administered the vapour of ether to sixteen different patients at the New York State Lunatic Asylum—viz., to fourteen men and to two women.

"Some have taken it but once, several have taken it three or four times, and a few eight or nine times.

"The cases in which we have used it have been various. Some were cases of melancholy and of religious despair, others were affected by various insane delusions and hallucinations, and some belonged to the demented class. To none highly excited or maniacal have we as yet given it.

"Some were not affected at all by it. One man and one woman inhaled it for more than ten minutes without experiencing the slightest change of feelings. Several seemed intoxicated, and said they felt as if drunk. One who had slept but little for several nights, and who usually slept poorly, rested remarkably well the night after taking it, and said he must have taken a large dose of opium.

"Some have appeared better since they commenced taking it, been more active, cheerful, and sociable. One who has taken it nine times seems considerably improved. He was previously dull, inactive, and unsocial, and his pulse but 48 in a minute. Since the use of the ether, his pulse has increased to 66 in a minute. He is now cheerful and sociable, and works some. He says he is better, and thinks the ether has benefited him.

"A few were highly excited by it. One man who was in a state of religious despair, after taking it, awoke as from a terrific dream, and in a most violent rage seized the person who administered the ether. He afterwards said that he at first dreamed he was in hell, and that taking the ether had sent him there, and hence his rage and violence against the operator.

"When this excitement abated, he seemed ecstatic with delight on account of the visions he had seen, and the revelations that had been made to him. 'I floated away,' he exclaimed, 'in infinity of space; I have seen a future world; what I have seen has proved the dogmas of religion; unless a man comes up to an iota, it is over with him.' He said he felt 'convinced of the truth of Newton's theory of the solar system, as he saw the planets revolving in the order and way pointed out.' When fully recovered from the effects of the ether, he recollected the assault and begged forgiveness.

"Some were pleasantly excited after using it. One danced. Another, when asked how he felt after awaking from a short sleep, replied, 'exactly, exactly neat, by jingo—I never felt better in my life than I do now. I thought I was in heaven, then in hell, then at the judgment, and then at school; I must have slept two hours.' Another, when asked by a patient to tell him what his feelings were, said, 'he felt like a kind of airy nothingness, as if he could fly.' To none has it proved the least injurious, and we are rather favourably impressed with its use, though we do not expect any striking remedial effects from it. We shall, however, continue our inquiries, and shall endeavour to ascertain if there is not some class of the insane to whom it is especially useful."

In the early part of last year [February, 1848], two of the managers of the New York State Lunatic Asylum and party journeyed by land, or by the mail route, from Utica to New Orleans, and returned by the Mississippi and Ohio rivers, visiting on their way the institutions for the insane which lay within their route. In the journal before us, the notes, relating to these institutions, taken by one of the travellers, have been given under the head of an article, entitled, "Editorial Correspondence," which is extremely amusing and interesting:—

"Upon our way to Washington," observes our traveller, March 2, 1848, "we passed two days at Albany, where we found much to interest us. The State Normal School, the Geological Rooms, the Capitol and State Library, are very deserving the attention of the traveller. The state library, which is in the capitol, is a very valuable and admirably-arranged collection of books. While in the Law Library we had the curiosity to look up some of the oldest laws relating to the insane in the state of New York, but we found no provision for their cure and comfortable maintenance, but merely for their safe-keeping, that they might not endanger others, and for the preservation of their property. If we mistake not, there is no allusion to the insane, with reference to their restoration, in any of the laws of the state, until the act to 'organize the State Lunatic Asylum, and more effectually to provide for the care, maintenance, and recovery of the insane,' was passed, April 7, 1842. In some of the oldest laws, the insane are denominated persons of 'unsane memory.' We were much pleased with the additions to the State Library that have been obtained through the exertions of M. Vattemare. Among the French works we saw some relating to insanity. One large volume of the 'State Trials of France' is devoted to the trial of Joseph Henri for firing at the king, Louis Philippe, July, 1846, and whom our readers will recollect we supposed to have been insane. See *Journal of Insanity*, vol. ii. p. 184. On looking over the account of his trial, it seems to us he was not defended with the zeal and ability he ought to have been. He was found guilty and condemned to the galleys for life, but we have seen it stated in the newspapers that since the late revolution in France he has been set at liberty. The State Library does not contain many distinct works on insanity, but there is in Albany one of the best collections of books on this subject, belonging to Dr. T. R. Beck, that we have ever seen.

"We made no stay in New York, but passed a day in Philadelphia, and visited the Pennsylvania Hospital for the Insane, under the care of Dr. Kirkbride. We found this establishment as usual in excellent order, and noticed some late improvements. We were particularly desirous of seeing the detached cottage that had recently been erected for patients. It is a neat building, one story high, 46 feet by 25, and placed about 40 feet from the main hospital, and adjoining the ladies' yard. It was occupied by two females, and seemed to us to be a very desirable appendage to a large establishment for the insane.

"We were pleased to learn that preparations were in progress for establishing a museum at this hospital. The one at the New York Asylum, at Utica, though small as yet, has proved to be a source of interest and gratification to many patients, both while at the institution and after they have returned to their homes. In a previous number of this journal, we suggested that a museum, or collection of minerals, shells, pictures, specimens of ancient and modern art, and curiosities of all sorts, should be connected with institutions for the insane; and we are pleased to learn that several have commenced making preparations for the purpose. The day we were at the hospital, Dr. Curwen, the excellent assistant-physician, was making preparations for delivering a lecture, on some branch of natural history, to the patients in the evening. This, as a means of benefiting the insane, we think highly of, and have long urged increased attention to the exercise of the mental faculties in many cases of insanity, especially in monomania and dementia, as a remedial measure, and one founded on the pathology of the disease. We wish, in addition to lectures, that schools should be established in every lunatic asylum, and considered an essential part of an ameliorating and curative plan of treatment. Attention to geography, history, arithmetic, natural philosophy, composition, drawing, &c., under the care of a competent and judicious teacher, who acts under the orders of the medical superintendent, leads the patient to exercise the partially atrophied, bloodless, or else congested organs of faculties that have long been dormant; and this, we think, in many cases, is essential to improvement. It is not always enough to be mere listeners to what others say, or passive recipients of the ideas of others; but the patient should be induced by kind and persevering attention to exert his own mental powers, and thus 'minister to himself.' The mind thus becomes invigorated, and not unfrequently the circulation; and the general health is improved, and a desire created for bodily exertion."

Arrived at Washington, the travellers discover, to their surprise, that there is no asylum for the insane, who are for the most supported by public charge at the Maryland Hospital, Baltimore. After visiting many places of interest at Washington, particularly the Senate and House of Representatives, the Supreme Court, the Congressional Library, Patent Office, and calling on the heads of departments, attending the levee at the president's, the travellers left Washington, and passing through Virginia, North Carolina, South Carolina, Georgia, and Alabama, arrived at New Orleans. There are two State Lunatic Asylums in Virginia—one at Williamsburg and one at Staunton. There are none in North Carolina; but one at Columbia, South Carolina, and one at Milledgeville, Georgia. Upon arriving at Mobile, our travellers had an interesting conversation with Dr. Nott, distinguished by his writings on "The Natural History of the Caucasian and Negro Races." He is of opinion [observes our note-taker] that—

"That the white and negro races are distinct species, and inclined to consider the mulattoes as hybrids, a degenerate unnatural offspring, doomed by nature to work out its own destruction. He says:

"1st. That the mulattoes are intermediate in intelligence between the blacks and whites.

"2nd. That they are less capable of undergoing fatigue and hardships than the blacks or whites.

"3rd. That the mulatto women are particularly delicate, and subject to a variety of chronic diseases.

"4th. That the women are bad breeders and bad nurses; many do not conceive; most are subject to abortions, and a large portion of the children die young in the southern states.

"5th. That the two sexes, when they intermarry, are less prolific than when crossed on one of the parent stocks.

"6th. That negroes and mulattoes are exempt, in a surprising degree, from yellow fever.

"He says the mulattoes, derived from the mixture of the Spanish or French with the negro, are more robust, finer-looking, more prolific, and longer-lived than those from the union of the Anglo-

Saxon race and negro. He thus accounts for the healthy appearance, fine forms, and agreeable countenances of many of the coloured creoles of Mobile and New Orleans."

Arrived at New Orleans, they found much to attract the attention of travellers, but little to interest respecting the insane. There was, in fact, no state asylum for their reception, although one was building at Jackson, about one hundred and fifty miles above New Orleans. The insane of the state were kept in a building in the rear of the Charity Hospital; and the following graphic account of the inconveniences attending the inadequate provision for the insane needs no comment:—

"At the time of our visit, there were about seventy insane patients, mostly old and demented cases, under the care of the house-surgeon, Dr. Wedderstrandt, who devotes himself with benevolent zeal to their comfort and welfare. Many of these assist about the hospital; but it is a poor place for the insane. They are annoyed and injured by the patients in the General Hospital, and there are no suitable grounds for exercise. We saw one man walking in a small yard with iron fetters on, to prevent his running away. We noticed that many slept in dormitories with musquito nets attached to the bedsteads. The irritation occasioned by musquitoes in this region must be great to those who are so deranged that they cannot make use of such nets. We saw here a padded room. Notwithstanding the pads were made of very strong cloth, it had recently been torn by a violent patient, and the hair scattered about the room. A very large proportion of the admissions into the lunatic department are cases of mania a potu. The total number of admissions into this department, including cases of mania a potu, for 1847, was 678. Discharges, 541; deaths, 25.

"Dr. W. told us that Indians, though drunk half of the time, did not have mania a potu; and he thinks the Spanish, French, and the negroes, when exposed to the same causes, far less liable to this disease than the English, Irish, and Germans.

"The reception of patients into the General Hospital has been very great the past year—viz., 11,690; of which number 9369 were discharged; 2037 died; and 828 remained January 1st, 1848.

"One hundred patients were received in one day, nearly all of whom were foreigners—mostly Irish, suffering from ship fever. Many of the medical and other assistants suffered from fever thus introduced. Twenty of the Sisters of Charity were affected by it, seven of whom died. Ten of the medical students, who acted as assistants, had the fever, and remained in the hospital; but none died. . . .

"We visited the United States Barracks, now mainly a hospital for sick and wounded soldiers, under the care of Dr. Wood, to whom we were indebted for valuable information and many civilities. He is the son-in-law of General Taylor, and has long been connected with the army, and now resides with his family at the barracks. We here saw some of the sad results of war. Men of robust frames broken down by long-continued disease, caused by irregular diet, change of habits, and exposure in a bad climate; others with loss of limbs, and suffering from severe wounds. We also saw here two deranged soldiers; one was probably insane when he enlisted. We understood that application had been made for their discharge, and that probably they would soon be removed. We have known four instances of insane persons enlisting. Two were soon discharged; but two others, who had been patients here, and not fully restored when they left and enlisted, have served two or three years in the army. We have had several letters from them. One complains much of his hardships, and wishes to be discharged; the other seems delighted with the opportunity afforded him of seeing new countries, and makes no complaint."

Leaving New Orleans, the travellers proceeded by steam down the Missouri, in St. Louis, a distance of 1230 miles. They found no state lunatic asylum in Missouri, but one building at Fulton, Galloway County, near the centre of the state. But whether by steamboat or railway, we cannot afford space to continue *en route;* and therefore take farewell of our travellers at Columbus, where we are informed that the Ohio Lunatic Asylum is highly creditable to the state.

We had marked numerous articles in the American *Journal of Insanity* for analysis, and a variety of passages for quotation. We have, in fact, been perplexed by an *embarras de richesses;* but we shall have frequent occasion to return to interesting matters contained in these volumes; and in the meantime, we must avow that to Dr. Brigham and his able coadjutors, the medical profession, on this as well as on the other side of the Atlantic, are deeply indebted. To the Association of Medical Superintendents connected with institutions for the insane in America, we are also under manifest obligation. To the conjoint labours of such men, animated by a spirit of independence and true love of science, we look forward with the highest feeling of interest; their researches will, we doubt not, advance our knowledge of the pathology and treatment of insanity, and throw light on the most obscure of all sciences—that which we designate in modern language, the science of psychology.

